# Supplementary material for: Are carbon emissions trading and green financial instruments synergistic? -Comprehensive quantitative research based on content analysis
Source: PLoS One. 2024 Mar 7;19(3):e0298601. doi: 10.1371/journal.pone.0298601 (PMC10919601; doi:10.1371/journal.pone.0298601)
Supplement: S1 Data — (ZIP) [file pone.0298601.s001.zip › Data/地级市Z_F工作报告.zip!/║■─╧/╘└╤⌠╩╨/└┤╫╘╣1⁄2╓┌║┼í╛┬φ┐╦╩2╛▌═°í┐_2006.docx]

2006年是我国实施“十一五”规划并实现良好开局的一年

——经济平稳快速增长。国内生产总值20.94万亿元，比上年增长10.7%；居民消费价格总水平上涨1.5%。经济增长连续四年达到或略高于10%，没有出现明显通货膨胀。

——经济效益稳步提高。全国财政收入3.93万亿元，比上年增加7694亿元；规模以上工业企业实现利润增长31%，增加4442亿元。

——改革开放进一步深化。重点领域和关键环节改革取得新进展。进出口贸易总额1.76万亿美元，比上年增长23.8%；实际利用外商直接投资695亿美元。

——社会事业加快发展。科技创新取得重大成果，教育事业继续发展，公共卫生体系建设得到加强，文化、体育事业进一步繁荣。

——人民生活有较大改善。城镇新增就业1184万人。城镇居民人均可支配收入11759元，农村居民人均纯收入3587元，扣除价格因素，分别比上年实际增长10.4%和7.4%。

这些成就，标志着我国综合国力进一步增强，我们朝着全面建设小康社会目标又迈出坚实的一步。

总结我们的实践经验，归结起来就是，只有解放思想、实事求是，与时俱进、开拓创新，坚定不移地走中国特色社会主义道路，坚持改革开放，坚持科学发展、和谐发展、和平发展，才能最终实现现代化的宏伟目标。

过去一年主要做了七个方面的工作

一、加强和改善宏观调控。针对经济运行中投资增长过快、货币信贷投放过多、外贸顺差过大等突出问题，中央及时采取了一系列宏观调控措施。加强土地调控，从紧控制新增建设用地，严肃查处违法违规用地行为；加强货币信贷管理，两次上调人民币贷款基准利率，三次上调金融机构存款准备金率；加强财政、税收对经济运行的调节；加强新上项目市场准入审核和监督检查；加强房地产市场调控和监管，着力调整住房供给结构。宏观调控措施逐步见效，固定资产投资增幅回落，银行信贷投放增速放缓，防止了经济增长由偏快转为过热，避免了经济大起大落。

二、加大“三农”工作力度。稳步推进社会主义新农村建设。全年中央财政用于“三农”支出3397亿元，比上年增加422亿元。在全国范围内取消了农业税和农业特产税，终结了延续2600多年农民种田交税的历史。继续增加对种粮农民直接补贴、良种补贴和农机具购置补贴，实施农业生产资料综合补贴政策，继续对重点地区的重点粮食品种实行最低收购价政策，增加对财政困难县乡和产粮大县的转移支付。全年粮食产量达到9949亿斤，实现了连续三年增产。农村道路、水利、电力、通信等基础设施建设得到加强，又有2897万农村人口解决了安全饮水问题，新增450万农村沼气用户，农民的生产生活条件继续改善。农村扶贫开发工作取得新进展。制定并实施解决农民工问题的政策措施，从解决工资偏低和拖欠、依法规范劳动管理、加强就业培训和社会保障等方面，维护了农民工的合法权益。

三、加快经济结构调整。制定了加快振兴装备制造业的政策措施，推进关键领域重大技术装备自主制造。开展百万千瓦级核电机组、超临界火电机组、新型船舶等设备自主化工作，启动高档数控机床和重要基础制造装备等重大专项。制定并实施钢铁、煤炭、水泥等11个行业结构调整的政策措施，部分产能过剩行业投资增幅明显回落，新开工项目计划总投资大幅下降，煤炭、电解铝分别淘汰落后生产能力1.1亿吨和120万吨。一批关系国民经济全局的重大工程建成投产或开工建设。青藏铁路全线通车，三峡左岸电站机组全部建成发电，金沙江向家坝水电站开工建设。这些对增强经济发展后劲将发挥重要作用。

更加重视节能环保工作。完善节能降耗、污染减排政策，普遍建立节能减排目标责任制。积极推进重点行业、重点企业和重点工程的节能工作，开展循环经济试点，加强三峡库区、松花江、南水北调水源及沿线、渤海等重点流域和区域水污染防治工作，实施燃煤电厂脱硫、城市污水处理、垃圾无害化处理等环保重点工程。连续四年在全国开展整治违法排污企业、保障群众健康环保专项行动。扎实推进生态保护和建设。整顿规范矿产资源开发秩序取得阶段性成果。地质勘查工作得到加强。

四、积极推进改革开放。全面部署农村综合改革。集体林权制度改革稳步进行。国有经济布局调整、国有企业股份制改革和国有资产监管取得新进展。邮政体制实现政企分开，电力体制改革继续推进。国有商业银行股份制改革迈出新的重要步伐，中国银行、中国工商银行在境内外成功上市，农村信用社改革取得阶段性成果；上市公司股权分置改革基本完成，证券市场基础性制度建设得到加强；保险业改革深入推进；外汇管理体制改革逐步深化。财政税收制度继续完善。公务员工资制度改革和规范收入分配秩序顺利进行。整顿和规范市场秩序工作深入开展。

调整对外贸易结构。完善出口退税、关税和加工贸易政策，控制高耗能、高污染产品出口；积极增加进口。优化利用外资结构，扩大银行、分销、电信等服务业对外开放。深化和扩大与各国的经贸往来。支持企业对外投资合作。全面履行加入世界贸易组织承诺，完善涉外经济体制和法规政策。

五、大力发展社会事业。全年中央财政用于科技、教育、卫生和文化事业的支出分别为774亿元、536亿元、138亿元和123亿元，比上年增长29.2%、39.4%、65.4%和23.9%。

推进科技创新。历时两年研究制定的国家中长期科学和技术发展规划纲要颁布实施，并制定了相关专项规划和配套政策措施。大型油气田和煤层气开发、新一代宽带无线移动通信网、大型飞机设计与制造、载人航天与探月工程等16个重大专项陆续启动。高性能超级计算机、超级优质杂交水稻、第三代移动通信、数字电视等一些关键技术取得突破，自主创新能力增强。

优先发展教育事业。全国财政安排农村义务教育经费1840亿元，全部免除了西部地区和部分中部地区农村义务教育阶段5200万名学生的学杂费，为3730万名贫困家庭学生免费提供教科书，对780万名寄宿学生补助了生活费。410个“两基”（基本普及九年义务教育和基本扫除青壮年文盲）攻坚县已有317个县实现目标，西部地区“两基”人口覆盖率由2003年的77%提高到96%。中央财政连续三年累计投入90亿元，用于农村寄宿制学校建设工程，7651所学校受益。农村中小学现代远程教育工程已投入80亿元，覆盖中西部地区80％以上的农村中小学，1亿多中小学生得以共享优质教育资源。

加强医疗卫生工作。覆盖城乡、功能比较完善的疾病预防控制体系和突发公共卫生事件医疗救治体系，已经基本建成。启动了农村卫生服务体系建设，中央财政安排27亿元国债资金用于县、乡、村三级医疗卫生基础设施建设。新型农村合作医疗试点范围扩大到1451个县（市、区），占全国总数的50.7%，有4.1亿农民参加；中央财政支出42.7亿元，地方财政也相应增加支出，较大幅度提高参加合作医疗农民的补助标准。以社区为基础的城市医疗服务体系建设加快推进。城乡医疗救助工作有所加强。中央财政安排51亿元，用于支持地方加强公共卫生服务，艾滋病等重大疾病防控取得明显进展。

积极发展文化、体育事业。新闻出版、广播影视、文学艺术、哲学社会科学进一步繁荣。文化基础设施尤其是农村基层文化设施建设得到加强，广播电视村村通工程由行政村向自然村延伸，社区和乡镇综合文化站工程、全国文化信息资源共享工程建设继续推进。

六、努力做好就业和社会保障工作。进一步完善和落实就业再就业扶持政策，中央财政安排就业再就业资金234亿元。多渠道开发就业岗位，加强职业技能培训工作，开展对“零就业家庭”和就业困难人员的帮扶，505万下岗失业人员实现再就业。2004年我们提出用三年时间基本解决建设领域历史上拖欠工程款和农民工工资的问题，这项工作基本完成，各地已偿还拖欠工程款1834亿元，占历史拖欠的98.6%，其中清付农民工工资330亿元。

加强社会保障体系建设。在东北三省试点的基础上，企业职工基本养老保险做实个人账户试点范围又扩大了8个省份。经过多年努力，国有企业下岗职工基本生活保障向失业保险并轨基本完成。提高了企业离退休人员基本养老金标准。社会保险覆盖面继续扩大，社会保险基金收入不断增加，社保基金使用的监督管理得到加强。城乡社会救助体系框架基本建立，慈善事业不断发展。中央财政用于城市低保的补助资金136亿元，比上年增加24亿元，各地不同程度提高了城市低保补助水平。已有25个省（自治区、直辖市）、2133个县（市、区）初步建立了农村最低生活保障制度，1509万农民享受了农村最低生活保障。基本实现了农村“五保”从农民集体互助共济向财政供养为主的转变。加强了孤儿救助和流浪未成年人保护工作。大幅度提高各类优抚对象的抚恤补助标准，全年中央财政抚恤事业费支出112亿元，比上年增长47%。完善大中型水库征地补偿和移民后期扶持政策，扶持对象达2288万人，这个长期遗留的问题正在逐步解决。

七、继续加强民主法制建设。基层民主政治建设不断推进。政府立法工作进一步加强。国务院向全国人大常委会提请审议企业所得税法草案、反垄断法草案、突发事件应对法草案和义务教育法修订草案等7件法律议案，颁布了艾滋病防治条例、外资银行管理条例等29个行政法规。加快建设法治政府，认真贯彻行政许可法、公务员法和全面推进依法行政实施纲要。监察、审计监督工作力度加大。司法行政体制和工作机制改革稳步推进。信访工作得到加强。社会治安防控体系不断完善，平安创建活动广泛开展。集中整治治安混乱地区和突出治安问题取得明显成效。廉政建设和反腐败斗争深入推进，开展治理商业贿赂专项工作，严肃查处了一批涉及政府机关及其工作人员的大案要案，依法惩处了一批腐败分子。

民族、宗教、港澳、对台和侨务工作进一步加强。国防和军队现代化建设取得新进展。外交工作成绩显著。

经济社会发展中的矛盾和问题

我国经济社会发展中仍然存在不少矛盾和问题，政府工作还有一些缺点和不足。

一是经济结构矛盾突出。一二三产业比例不合理，城乡之间、地区之间发展不平衡，投资消费关系不协调。农业基础薄弱状况没有改变，粮食稳定增产和农民持续增收难度加大。固定资产投资总规模依然偏大，银行资金流动性过剩问题突出，引发投资增长过快、信贷投放过多的因素仍然存在。外贸顺差较大，国际收支不平衡矛盾加剧。

二是经济增长方式粗放。突出表现在能源消耗高、环境污染重。“十一五”规划提出了节能降耗和污染减排目标，并作为约束性指标。一年来，各地区各部门做了大量工作，取得了积极进展。2006年，单位国内生产总值能耗由前三年分别上升4.9%、5.5%、0.2%，转为下降1.2%；主要污染物排放总量增幅减缓，化学需氧量、二氧化硫排放量由上年分别增长5.6%和13.1%，减为增长1.2%和1.8%。但是，全国没有实现年初确定的单位国内生产总值能耗降低4％左右、主要污染物排放总量减少2%的目标。主要原因是：产业结构调整进展缓慢，重工业特别是高耗能、高污染行业增长仍然偏快，不少应该淘汰的落后生产能力还没有退出市场，一些地方和企业没有严格执行节能环保法规和标准，有关政策措施取得明显成效需要一个过程。“十一五”规划提出这两个约束性指标是一件十分严肃的事情，不能改变，必须坚定不移地去实现，国务院以后每年都要向全国人大报告节能减排的进展情况，并在“十一五”期末报告五年这两个指标的总体完成情况。

三是一些涉及群众利益的突出问题解决得不够好。食品药品安全、医疗服务、教育收费、收入分配、社会治安、安全生产等方面还存在群众不满意的问题，土地征收征用、房屋拆迁、企业改制、环境保护等方面损害群众利益的问题仍未能根本解决。不少低收入群众生活比较困难。

四是政府自身建设存在一些问题。政府职能转变滞后，政企不分依然存在，有些部门职责不清，办事效率低；公务消费不规范，奢侈浪费，行政成本高；一些地方、部门和少数工作人员还存在官僚主义、形式主义，脱离群众，失职渎职，甚至滥用权力，贪污腐败。存在这些问题，根本在于制度不健全、监管不到位。

我们必须以对国家和人民高度负责的精神，采取更加有力的措施，切实解决这些问题，决不辜负人民的期望。

2007年政府工作的基本思路和主要目标

2007年政府工作的基本思路和任务是：以邓小平理论和“三个代表”重要思想为指导，全面落实科学发展观，加快构建社会主义和谐社会，认真贯彻党的十六大以来各项方针政策，加强和改善宏观调控，着力调整经济结构和转变增长方式，着力加强资源节约和环境保护，着力推进改革开放和自主创新，着力促进社会发展和解决民生问题，全面推进社会主义经济建设、政治建设、文化建设、社会建设，为党的十七大召开创造良好的环境和条件。

综合考虑各种因素，今年国民经济和社会发展的主要目标是：在优化结构、提高效益和降低消耗、保护环境的基础上，国内生产总值增长8％左右；城镇新增就业人数不低于900万人，城镇登记失业率控制在4.6%以内；物价总水平基本稳定，居民消费价格总水平涨幅在3%以内；国际收支不平衡状况得到改善。

提出今年国内生产总值增长8％左右的目标，综合考虑了需要和可能等多种因素，更重要的是要引导各方面认真落实科学发展观，把工作重点放到优化结构、提高效益、节能降耗和污染减排上来，防止片面追求和盲目攀比增长速度，实现经济又好又快发展。

今年政府工作必须把握好五个政策原则

实现今年经济社会发展的目标和任务，必须把握好五个政策原则。

一是稳定、完善和落实政策。稳定，就是保持宏观经济政策的连续性和稳定性，继续实施稳健的财政政策和货币政策。完善，就是根据经济运行新情况及时完善政策措施，有针对性地解决突出矛盾和问题。落实，就是认真落实中央的各项政策措施，增强执行力，真正把各项决策部署落到实处。

二是加强和改善宏观调控。重点是控制固定资产投资和信贷规模，在优化结构中促进经济总量平衡。坚持有保有压，不搞一刀切。更多地运用经济杠杆、法律手段引导和规范经济行为。正确处理中央和地方的关系，充分发挥两个积极性。

三是大力提高经济增长质量和效益。把节约能源资源、保护环境、集约用地放在更加突出的位置，注重提高产品质量，增强经济竞争力和可持续发展能力。

四是更加重视社会发展和改善民生。坚持以人为本，促进社会事业加快发展，积极解决人民群众最关心、最直接、最现实的利益问题，维护社会公平正义，让全体人民共享改革发展成果。

五是以改革开放为动力推进各项工作。坚持社会主义市场经济改革方向，适应经济社会发展要求，推进经济体制、政治体制、文化体制、社会体制改革，加快构筑落实科学发展观和建设和谐社会的体制保障。全面提高对外开放水平。

要坚持加强和改善宏观调控

促进经济又好又快发展，要坚持加强和改善宏观调控。

要继续实行稳健的财政政策。一是适当减少财政赤字和长期建设国债规模。今年中央财政赤字拟安排2450亿元，比去年预算赤字减少500亿元；拟安排长期建设国债500亿元，比去年减少100亿元；拟安排中央预算内经常性建设投资804亿元，比去年预算增加250亿元；中央政府建设投资总规模为1304亿元。二是政府预算支出和政府投资要优化结构、突出重点。政府投资使用要确保“三个高于”，即：用于直接改善农村生产生活条件的投入高于上年，用于基础教育和公共卫生等社会事业的投入高于上年，用于西部大开发的投入高于上年。同时，加大对节能环保和自主创新的支持。三是合理安排中央财政超收收入。近几年，由于实际经济增长高于预期目标，加上一些政策性增收因素，中央财政超收较多。超收收入主要用于解决出口退税和退耕还林等历史欠账，依法增加对地方税收返还和一般转移支付，增加教育、科技等法定支出，增加社会保障基金、企业政策性破产、居民最低生活保障等支出。今年拟从去年超收收入中安排500亿元设立中央预算稳定调节基金。这样做，对于更加科学合理地编制预算，保持中央预算的稳定性和财政政策的连续性，是完全必要的。这个基金的使用纳入预算管理，接受全国人大监督。

继续实行稳健的货币政策。综合运用多种货币政策工具，合理调控货币信贷总量，有效缓解银行资金流动性过剩问题。调整和优化信贷结构，引导银行加大对“三农”、中小企业、节能环保和自主创新的信贷支持；继续控制中长期贷款，严格限制对高耗能、高污染企业和产能过剩行业的劣质企业贷款。稳步推进利率市场化改革。进一步完善人民币汇率形成机制。加强和改进外汇管理，积极探索和拓展国家外汇储备合理使用的渠道和方式。从多方面采取措施，逐步改善国际收支不平衡状况。

调整投资与消费的关系。坚持扩大内需方针，重点扩大消费需求。要采取多种措施，努力增加城乡居民收入特别是中低收入者的收入。合理调整和严格执行最低工资制度，落实小时最低工资标准，加强企业工资分配调控和指导，建立健全工资正常增长机制和支付机制。今年各地要对最低工资制度和小时最低工资标准的执行情况，普遍进行一次检查。继续落实公务员工资制度改革、规范公务员收入分配秩序及调整相关人员待遇的政策措施。特别要扩大农村消费需求，认真落实促进农民增收减负的政策措施，加强农村商贸流通和市场体系建设，改善农村消费环境和条件。

要保持固定资产投资适度增长，着力优化投资结构，提高投资效益。继续严把土地、信贷闸门，根据不同行业情况，适当提高并严格执行建设项目用地、环保、节能、技术、安全等市场准入标准。严格控制新上项目，特别要控制城市建设规模。加强关系经济社会全局和长远发展的重大项目建设，加快大型水利、能源基地、铁路干线、国道主干线等重要基础设施建设。积极引导社会资金更多地投向农业农村、社会事业、自主创新、资源节约、环境保护和中西部地区。要深化投资体制改革，完善重大投资项目决策制度和责任追究制度。

要继续加强房地产市场调控和管理

房地产业对发展经济、改善人民群众住房条件有着重大作用，必须促进房地产业持续健康发展。一是从我国人多地少的国情和现阶段经济发展水平出发，合理规划、科学建设、适度消费，发展节能省地环保型建筑，形成具有中国特点的住房建设和消费模式。二是房地产业应重点发展面向广大群众的普通商品住房。政府要特别关心和帮助解决低收入家庭住房问题。加大财税等政策支持，建立健全廉租房制度。改进和规范经济适用房制度。三是正确运用政府调控和市场机制两个手段，保持房地产投资合理规模，优化商品房供应结构，加强房价监管和调控，抑制房地产价格过快上涨，保持合理的价格水平。四是深入整顿和规范房地产市场秩序，强化房地产市场监管，依法惩治房地产开发、交易、中介等环节的违法违规行为。地方各级政府要对房地产市场的调控和监管切实负起责任。

要以加快发展现代农业为重点，扎实推进社会主义新农村建设

做好今年的“三农”工作，要以加快发展现代农业为重点，扎实推进社会主义新农村建设。

一是稳定发展粮食生产。二是切实提高农业综合生产能力。三是大力加强农村基础设施建设。四是多渠道增加农民收入。五是着力推进农村实用人才队伍建设和农村人力资源开发。

发展现代农业，推进新农村建设，要靠政策、靠投入、靠科技、靠改革。一要巩固、完善和加强支农惠农政策。增加对种粮农民的直接补贴、良种补贴、农机具购置补贴和农业生产资料综合补贴。继续实行粮食最低收购价政策。进一步加大对财政困难县乡和产粮大县的支持力度。二要加大对农业农村投入力度。切实把国家基础设施建设和社会事业发展的重点转向农村，今年财政支农投入的增量要继续高于上年，国家固定资产投资用于农村的增量要继续高于上年，土地出让收入用于农村建设的增量要继续高于上年。中央财政安排用于“三农”的资金3917亿元，比去年增加520亿元。积极发展农业保险，扩大农业政策性保险试点范围。三要加快农业科技进步。加强农业科技创新能力建设，支持农业科技项目，加快农业科技成果转化，完善基层农业技术推广和服务体系，鼓励农业科技进村入户。四要全面推进农村综合改革。加快乡镇机构改革、农村义务教育改革和县乡财政管理体制改革，逐步建立精干高效的农村行政管理体制、政府保障的农村义务教育体制、覆盖城乡的公共财政制度。同时，推进征地制度、集体林权制度改革，积极发展农民专业合作组织，继续清理和化解乡村债务。

推进社会主义新农村建设，必须把重点放在发展农村经济、增加农民收入上。坚持稳定和完善农村基本经营制度，坚持因地制宜、从实际出发，坚持尊重农民意愿、维护农民权益，反对形式主义和强迫命令。

要大力抓好节能降耗、保护环境

今年在节能环保方面，重点要做好以下八个方面工作：一是完善并严格执行能耗和环保标准。新上项目必须进行能源消耗审核和环境影响评价，不符合节能环保标准的不准开工建设，现有企业经整改仍不达标的必须依法停产关闭。二是坚决淘汰落后生产能力。“十一五”期间，关停5000万千瓦小火电机组，今年要关停1000万千瓦；五年淘汰落后炼铁产能1亿吨、落后炼钢产能5500万吨，今年力争分别淘汰3000万吨和3500万吨。加大淘汰水泥、电解铝、铁合金、焦炭、电石等行业落后产能的力度。三是突出抓好重点行业和企业。加强钢铁、有色金属、煤炭、化工、建材、建筑等重点行业，以及年耗能万吨标准煤以上重点企业的节能减排工作。全面实施低效燃煤工业锅炉（窑炉）改造、区域热电联产等重点节能工程。坚持优先发展城市公共交通。四是健全节能环保政策体系。注重发挥市场机制作用，综合运用价格、财税、信贷等经济手段，促进节能环保工作。深化重要资源性产品价格和排污收费改革，完善资源税制度，健全矿产资源有偿使用制度。五是加快节能环保技术进步。积极推进以节能减排为主要目标的设备更新和技术改造，引导企业采用有利于节能环保的新设备、新工艺、新技术。加强资源综合利用和清洁生产，大力发展循环经济和节能环保产业。六是加大污染治理和环境保护力度。增加国债资金和中央预算内资金，支持城镇生活污水、垃圾处理及危险废物处理设施建设。继续搞好“三河三湖”（淮河海河辽河、太湖巢湖滇池）、松花江、三峡库区及上游、南水北调水源及沿线等重点流域污染治理。禁止污染企业和城市污染物向农村扩散，控制农村面源污染。七是强化执法监督管理。建立更加有效的节能环保监督管理体系，坚决依法惩处各种违法违规行为。八是认真落实节能环保目标责任制。抓紧建立和完善科学、完整、统一的节能减排指标体系、监测体系和考核体系，实行严格的问责制。

要守住18亿亩耕地这条红线

节约集约用地，不仅关系当前经济社会发展，而且关系国家长远利益和民族生存根基。在土地问题上，我们绝不能犯不可改正的历史性错误，遗祸子孙后代。一定要守住全国耕地不少于18亿亩这条红线。坚决实行最严格的土地管理制度。

一要认真执行土地利用总体规划和年度计划。坚决控制建设占地规模，加强耕地特别是基本农田保护，禁止擅自将农用地转为建设用地。认真落实新修订的禁止类和限制类项目用地的规定，特别要禁止别墅类房地产开发、高尔夫球场、党政机关和国有企事业单位新建培训中心等项目用地。二要抓紧完善和严格执行节约集约用地标准，包括农村集体建设用地和宅基地，都要控制增量，盘活存量，提高土地利用效率和集约化程度。三要切实控制工业用地，坚决执行工业用地出让最低价标准。四要落实建设用地税费政策，规范土地出让收支管理，切实执行国有土地使用权出让收支全额纳入地方预算的规定。五要严格土地管理责任制。落实土地督察制度。对各类土地违法违规案件都要严肃查处。

要加快推进产业结构升级和自主创新

要坚持走新型工业化道路，着力优化产业结构。重点是大力发展服务业，提升工业层次和水平，继续推进国民经济和社会信息化。要从改革体制、加大投入、完善政策等方面，鼓励和支持服务业加快发展，尤其要发展物流、金融、信息、咨询、旅游、社区服务等现代服务业。要加快发展高新技术产业，振兴装备制造业，积极发展可再生能源，有序发展替代能源，广泛应用先进技术改造提升传统产业。加快产能过剩行业调整。在优化产业结构中，注重运用经济、法律手段，加强产业规划和政策的引导。

要围绕建设创新型国家，认真落实国家中长期科学和技术发展规划纲要提出的目标任务。实施国家重大科技专项，努力攻克一批事关国计民生和国家安全的核心关键技术，依托重点工程推进重大装备自主化建设，力争在重点优势领域取得突破。加强基础研究、前沿技术研究和社会公益研究。加快建立以企业为主体、市场为导向、产学研相结合的技术创新体系。完善自主创新激励机制，落实鼓励和支持自主创新的财税政策、金融政策和政府采购制度。积极发展创业风险投资。抓紧制定并实施国家知识产权战略，切实加强知识产权保护。继续实施全民科学素质行动计划。

要进一步推动区域协调发展

要坚持统筹兼顾、合理规划、发挥优势、落实政策，促进区域协调发展。

继续推进西部大开发，重点是加强基础设施、生态环境建设和发展科技教育，发展特色优势产业。抓紧制定后续政策，巩固和发展退耕还林、退牧还草成果。继续实施天然林保护、防沙治沙、石漠化治理等重点生态工程。积极推进东北地区等老工业基地振兴，重点是加大工业结构调整力度，搞好重要行业、企业的重组改造，推动装备制造业、原材料加工业、高新技术产业和农产品加工业发展，加强商品粮基地建设。加快资源枯竭型城市经济转型试点和采煤沉陷区治理。加快棚户区改造。中部地区，重点是继续加强粮食主产区生产能力和农产品加工转化能力建设，加强能源、重要原材料基地、综合交通运输体系建设，支持发展先进制造业和高新技术产业。东部地区，重点是推进产业结构优化升级，增强自主创新能力和国际竞争力，在改革开放和科学发展上走在前面。继续发挥经济特区、上海浦东新区作用，推进天津滨海新区等条件较好地区开发开放。

要加大国家对欠发达地区支持力度，鼓励发达地区对欠发达地区对口援助，促进革命老区、民族地区、边疆地区、贫困地区经济社会加快发展。做好库区发展和库区移民工作。

今年要在全国农村全面免除义务教育阶段学杂费

今年要在全国农村全部免除义务教育阶段的学杂费，使农村1.5亿中小学生家庭普遍减轻经济负担；继续对农村贫困家庭学生免费提供教科书并补助寄宿生活费。

要完善农村义务教育经费保障机制，不断提高保障水平。今年全国财政安排农村义务教育经费2235亿元，比去年增加395亿元。“十一五”时期中央财政将投入100亿元，实施农村初中学校改造计划，地方政府也要相应增加这方面的投入。同时，继续解决好城市困难家庭和农民工子女接受义务教育问题。今年还要确保全面完成西部地区“两基”攻坚计划和农村中小学现代远程教育工程。让所有孩子都能上得起学，都能上好学，我们一定能够实现这个目标。

要坚持把教育放在优先发展的战略地位

教育是国家发展的基石，教育公平是重要的社会公平。要坚持把教育放在优先发展的战略地位，加快各级各类教育发展。

优先发展教育的总体布局是，普及和巩固义务教育，加快发展职业教育，着力提高高等教育质量。

要完善农村义务教育经费保障机制，不断提高保障水平。要把发展职业教育放在更加突出的位置，使教育真正成为面向全社会的教育，这是一项重大变革和历史任务。重点发展中等职业教育，健全覆盖城乡的职业教育和培训网络。深化职业教育管理体制改革，建立行业、企业、学校共同参与的机制，推行工学结合、校企合作的办学模式。高等教育要以提高质量为核心，加快教育教学改革，相对稳定招生规模，加强高水平学科和大学建设，创新人才培养模式，优化人才培养结构，努力造就大批杰出人才。支持和规范民办教育发展，发挥社会力量办学的积极性。

促进教育发展和教育公平的两项重大措施

为了促进教育发展和教育公平，将采取两项重大措施：一是从今年新学年开始，在普通本科高校、高等职业学校和中等职业学校建立健全国家奖学金、助学金制度，为此中央财政支出将由上年18亿元增加到95亿元，明年将安排200亿元，地方财政也要相应增加支出；同时，进一步落实国家助学贷款政策，使困难家庭的学生能够上得起大学、接受职业教育。这是继全部免除农村义务教育阶段学杂费之后，促进教育公平的又一件大事。

二是在教育部直属师范大学实行师范生免费教育，建立相应的制度。这个具有示范性的举措，就是要进一步形成尊师重教的浓厚氛围，让教育成为全社会最受尊重的事业；就是要培养大批优秀的教师；就是要提倡教育家办学，鼓励更多的优秀青年终身做教育工作者。

今年卫生事业改革发展重点抓好四件事

要加快卫生事业改革和发展，着眼于建设覆盖城乡居民的基本卫生保健制度，今年重点抓好四件事。这四件事是：一是积极推行新型农村合作医疗制度。试点范围扩大到全国80%以上的县（市、区），有条件的地方还可以搞得更快一些。中央财政安排补助资金101亿元，比去年增加58亿元。落实农村卫生服务体系建设与发展规划，健全县、乡、村三级农村卫生服务网络，每个乡镇由政府办好一所卫生院，采取多种形式支持每个行政村设立一个卫生室，建设农村药品供应网和监督网，加强农村卫生队伍建设，努力让广大农民享有安全、有效、方便、价廉的医疗卫生服务。

二是加快建设以社区为基础的新型城市卫生服务体系。优化城市医疗卫生资源配置，重点发展社区卫生服务，落实经费保障措施，方便群众防病治病。中央财政对中西部地区给予适当支持。

三是启动以大病统筹为主的城镇居民基本医疗保险试点，政府对困难群众给予必要的资助。

四是做好重大传染病防治工作。今年扩大国家免疫规划范围，将甲肝、流脑等15种可以通过接种疫苗有效预防的传染病纳入国家免疫规划。在免费救治艾滋病、血吸虫病等传染病患者的基础上，扩大免费救治病种。为此，中央财政增加支出28亿元。温家宝说，要大力扶持中医药和民族医药发展，充分发挥祖国传统医药在防病治病中的重要作用。

为了更好地推进卫生事业改革发展，国务院已经组织力量抓紧制定深化医药卫生体制改革方案，努力解决好广大群众关心的看病就医问题。

要加快发展文化事业和文化产业

要加快发展文化事业和文化产业。要推进文化体制改革，完善文化产业政策。繁荣新闻出版、广播影视、文学艺术，发展哲学社会科学。着眼于满足人民群众文化需求，保障人民文化权益，逐步建立覆盖全社会的公共文化服务体系。突出抓好广播电视村村通工程、社区和乡镇综合文化站建设工程、全国文化信息资源共享工程、农村电影放映工程、农家书屋工程。继续建设一批国家重大文化工程。加强网络文化建设和管理。搞好文化遗产、自然遗产和档案保护。加强文化市场管理，坚持开展扫黄打非。

要加强就业和社会保障工作

要加强就业和社会保障工作，坚持把扩大就业放在经济社会发展的突出位置。要实施有利于促进就业的财政、税收和金融政策，积极支持自主创业和自谋职业。重点做好下岗失业和关闭破产企业人员再就业工作，积极帮助“零就业家庭”和就业困难人员就业，加强高校毕业生就业指导和服务，推进退役军人安置改革。发展和谐劳动关系，保障劳动者合法权益。

要加强社会保障体系建设。今年中央财政安排社会保障支出2019亿元，比去年增加247亿元。继续完善企业职工基本养老保险制度，推进做实个人账户扩大试点工作。健全城镇职工基本医疗保险和失业、工伤、生育保险制度。加快建立适合农民工特点的社会保障制度，重点推进农民工工伤保险和大病医疗保障工作。抓紧研究制定社会保险关系跨地区转移接续办法。进一步扩大社会保险覆盖面，特别要做好外资、私营等非公有制企业和城镇灵活就业人员的参保工作。

要完善城乡社会救助体系。健全城市居民最低生活保障制度、城乡医疗救助制度、城市生活无着的流浪乞讨人员救助制度。今年要在全国范围建立农村最低生活保障制度，这是加强“三农”工作、构建和谐社会的又一重大举措。各地要根据实际，合理确定低保对象范围和标准，中央财政对困难地区给予适当补助。在全国城乡建立最低生活保障制度，对于促进社会公平、构建和谐社会具有重大而深远的意义。继续落实好优抚政策，妥善解决优抚对象的实际困难。切实抓好防灾减灾救灾工作，妥善安排受灾群众的生产生活。支持慈善事业发展。要让城乡百姓特别是困难群众都能享受到公共财政的阳光。

要强化安全生产工作和整顿规范市场秩序

要强化安全生产工作和整顿规范市场秩序。要坚决遏制重特大安全事故发生，实现全国安全生产状况稳定好转。要认真贯彻“安全第一，预防为主，综合治理”的方针，完善安全生产体制，切实落实责任制，加大安全生产投入，加强安全生产教育和培训。要继续打好煤矿瓦斯治理和整顿关闭两个攻坚战。切实搞好重点行业和领域安全专项整治工作，加强学校和人员密集场所安全防范。要依法加强监管，严肃查处安全生产事故。

要坚持标本兼治，深入整顿和规范市场秩序。加快社会信用体系建设。完善市场管理制度，强化市场监管。依法打击制假售假、虚假广告、商业欺诈、传销和变相传销、偷逃骗税、走私贩私等违法活动。大力开展食品安全专项整治，全面整顿药品市场秩序，保障人民群众饮食和用药安全。

要推进社会主义民主法制建设

要推进社会主义民主法制建设。发展民主，健全法制，是社会主义制度的内在要求。构建和谐社会，最重要的是加强民主法制建设，促进社会公平正义。他提出，要积极稳妥地推进政治体制改革，加快中国特色的民主政治建设。完善人民的民主权利保障制度，保障人民依法管理国家事务、管理经济和文化事业、管理社会事务。加强城乡基层自治组织建设。扩大基层民主，完善政务公开、厂务公开、村务公开等制度，保证人民依法直接行使民主权利。各级政府要坚持科学民主决策，完善重大问题集体决策制度、专家咨询制度、社会公示和听证制度、决策责任制度，依法保障公民的知情权、参与权、表达权、监督权。

要全面推进依法行政。加强政府立法工作，重点是发展社会事业、健全社会保障、加强社会管理、节约能源资源、保护生态环境等方面的立法。加强和改善行政执法，落实行政执法责任制。执法部门要严格按照法定权限和程序行使权力、履行职责。进一步加强行政监督。各级政府及其工作人员都要带头遵守宪法和法律，严格依法办事。要自觉接受人民代表大会及其常委会的监督，接受人民政协的民主监督，认真听取民主党派、工商联、无党派人士和各人民团体的意见。接受新闻舆论和社会公众监督，支持监察、审计部门依法独立履行监督职责。深入开展普法教育，做好行政复议、法律服务和法律援助工作。继续推进司法体制改革，维护司法公正。

要维护社会安定和谐

要维护社会安定和谐。要把维护人民群众的合法权益，作为正确处理新时期社会矛盾、促进社会安定和谐的出发点和落脚点。健全利益协调机制、诉求表达机制、矛盾排查调处机制和权益保障机制。认真落实涉及群众利益的各项政策，依照法律和政策及时解决群众反映的问题，坚决纠正土地征收征用、房屋拆迁、企业改制、环境保护中损害群众利益的行为。各级政府工作人员特别是领导干部要深入基层，主动为群众排忧解难。加强社区建设，完善基层服务和管理网络。加强信访工作，健全信访工作责任制。加强社会治安防控体系建设，大力整治突出治安问题和治安混乱地区。依法打击刑事犯罪活动，维护国家安全和社会稳定。

要深化国有企业改革

要深化国有企业改革。一要按照有进有退、合理流动的原则，推动国有资本更多地向关系国家安全和国民经济命脉的重要行业和关键领域集中。推进企业调整重组，支持有条件的企业做强做大。二要推进国有大型企业股份制改革，健全公司法人治理结构、投资风险控制机制和内部监督管理机制，建立适应现代企业制度要求的选人用人和激励约束机制。三要完善国有资产监管体制。建立国有资本经营预算制度，规范国家与企业的分配关系。今年将进行国有资本经营预算编制试点。规范国有企业改制和国有产权转让行为，防止国有资产流失，维护职工合法权益。四要抓紧解决国有企业历史遗留问题。继续对长期亏损、资不抵债企业和资源枯竭矿山实施关闭破产，积极稳妥推进企业政策性破产工作。推进主辅分离、辅业改制和分离企业办社会职能。

要加快推进垄断行业改革。进一步放宽市场准入，引入竞争机制，实行投资主体和产权多元化。深化电力、邮政、电信、铁路等行业改革，稳步推进供水、供气、供热等市政公用事业改革。

要鼓励、支持和引导个体私营等非公有制经济发展

要鼓励、支持和引导个体私营等非公有制经济发展。要认真落实中央制定的各项政策措施。鼓励非公有制经济参与国有企业改革，进入公用事业、基础设施、金融服务以及社会事业等领域。完善金融、税收、技术创新等政策，改进对非公有制企业的服务。依法保护非公有制企业合法权益。加强对非公有制企业的引导和管理，促进企业依法经营。

要推进财税体制改革

要推进财税体制改革。现在统一内外资企业所得税，使内外资企业处于平等竞争地位，时机和条件已经成熟。这项改革涉及制定新的企业所得税法，《中华人民共和国企业所得税法（草案）》将提请本次大会审议。

要加快公共财政体系建设，完善财政转移支付制度，改革预算管理制度，制定全面实施增值税转型方案和措施，建立规范的政府非税收入体系。

加快金融体制改革的六项任务

今年加快金融体制改革的六项任务是：一要深化国有银行改革。巩固和发展国有商业银行股份制改革成果。推进中国农业银行股份制改革。深化政策性银行改革，重点进行国家开发银行改革。

二要加快农村金融改革。构建分工合理、投资多元、功能完善、服务高效的农村金融组织体系。充分发挥中国农业银行、中国农业发展银行在农村金融中的骨干和支柱作用，继续深化农村信用社改革，增强中国邮政储蓄银行为“三农”服务的功能。适当调整和放宽农村地区银行业金融机构准入政策，鼓励各类资本进入农村金融机构。探索发展适合农村特点的新型金融组织，加大农村金融产品和服务创新力度，积极解决农村贷款难问题。

三要大力发展资本市场。推进多层次资本市场体系建设，扩大直接融资规模和比重。稳步发展股票市场，加快发展债券市场，积极稳妥地发展期货市场。进一步加强市场基础性制度建设，推进股票、债券发行制度市场化改革，切实提高上市公司质量，加强市场监管。

四要深化保险业改革，扩大保险覆盖面，提高保险服务水平和防范风险能力。

五要推进金融对外开放，提高开放水平。

六要切实加强和改进金融监管，健全监管协调机制，有效防范和化解金融风险，维护国家金融稳定和安全。

要全面提高对外开放水平

要全面提高对外开放水平。要发展对外贸易。通过发展对外贸易促进经济发展、增加就业，是我们必须长期坚持的方针。要优化进出口结构，转变外贸增长方式，努力缓解外贸顺差过大的矛盾。支持具有自主品牌和高附加值产品出口，扩大服务产品和农产品出口，控制高耗能、高污染产品出口，促进加工贸易转型升级。要增加能源、原材料以及先进技术装备、关键零部件进口。加强国家口岸管理和检验检疫工作。

要做好利用外资工作。注重提高引进外资质量和优化结构，更多地引进先进技术、管理经验和高素质人才。引导跨国公司把高端制造和研发环节转移到我国，吸引外资加快向中西部、东北地区等老工业基地和符合产业政策的领域扩展。大力承接国际服务外包，提高我国服务业发展水平。加强对外资并购的引导和规范。优化投资环境，规范招商引资行为，纠正一些地方违法违规变相给予优惠政策和层层下达分解指标的做法。

要引导和规范企业对外投资合作。完善财税、信贷、外汇、保险等政策措施，支持有实力、有信誉、有竞争力的各种所有制企业走出去。加强引导和协调，避免企业在境外盲目投资和恶性竞争。发展对外承包工程与劳务合作。办好境外经济贸易合作区。推动多哈回合谈判进程，积极参与多边贸易规则制定，稳步推进双边和区域自由贸易区建设。

加强政府自身改革和建设的进展

总结了加强政府自身改革和建设的进展。做好新形势下的政府工作，必须不断加强政府自身改革和建设。几年来，我们把实行科学民主决策、推进依法行政、加强行政监督作为政府工作的三项基本准则，注重全面履行政府职能，着力加强社会管理和公共服务，建立健全应对突发公共事件管理体制机制，推进行政审批制度改革，制定和贯彻全面推进依法行政实施纲要，加强监察和审计工作，加大反腐倡廉力度。政府自身改革和建设迈出了重要步伐。

加强政府自身改革和建设，必须坚持以人为本、执政为民，把实现好、维护好、发展好最广大人民的根本利益作为出发点和落脚点；必须坚持从国情出发，实现党的领导、人民当家作主和依法治国的有机统一；必须坚持不断完善社会主义市场经济体制，促进经济社会全面协调可持续发展；必须坚持创新政府管理制度和方式，提高政府工作的透明度和人民群众的参与度。我们的目标是，建设一个行为规范、公正透明、勤政高效、清正廉洁的政府，建设一个人民群众满意的政府。

政府自身改革和建设今年集中抓好三项工作

当前和今后一个时期，要以转变政府职能为核心，规范行政权力，调整和优化政府组织结构与职责分工，改进政府管理与服务方式，大力推进政务公开，加快电子政务和政府网站建设，提升公务员队伍素质，全面提高行政效能，增强政府执行力和公信力。今年要集中力量抓好三项工作。

一是完善宏观调控体制，坚持政企分开，深入推进行政审批制度改革。二是加强社会管理和公共服务，增强基本公共服务能力，着力解决人民群众反映强烈的问题。三是依法规范行政行为，深入开展廉政建设和反腐败斗争，完善教育、制度、监督并重的惩治和预防腐败体系。

当前要解决一些行政机关存在的严重铺张浪费的问题

当前一个重要任务，就是解决一些行政机关存在的严重铺张浪费问题。现在，不少地方、部门和单位讲排场、比阔气，花钱大手大脚，奢侈之风盛行，群众反映强烈。这种不良风气必须坚决制止。要严格控制行政机关新建、扩建办公大楼，严禁建设豪华楼堂馆所，切实规范公务接待行为，堵塞管理漏洞，努力降低行政成本，建设节约型政府。

国防军队建设，是现代化建设的战略任务

建立巩固的国防，建设强大的人民军队，是社会主义现代化建设的战略任务。要坚持以毛泽东军事思想、邓小平新时期军队建设思想、江泽民国防和军队建设思想为指导，认真贯彻胡锦涛同志关于军队建设的一系列重要论述，全面落实科学发展观，着眼于有效履行新世纪新阶段军队的历史使命，坚持把思想政治建设放在首位，积极推进中国特色军事变革，确保部队高度稳定和集中统一，为维护国家安全统一和现代化建设提供坚强有力的保障。积极推进机械化条件下军事训练向信息化条件下军事训练转变，不断提高部队信息化条件下的防卫作战能力。加强国防科研和武器装备建设。推进后勤建设和改革。坚持依法治军、从严治军，提高军队正规化水平。加强人民武装警察部队全面建设，完成好执勤、处置突发事件、反恐斗争和维护稳定任务。深入开展国防教育，完善国防动员体制。搞好双拥共建活动，巩固和发展军政军民团结。

将为维护和发展香港、澳门繁荣稳定而努力奋斗

将与港澳各界人士一道，为维护香港、澳门长期繁荣稳定而努力奋斗。我们将继续坚定不移地贯彻“一国两制”、“港人治港”、“澳人治澳”、高度自治的方针，严格按照特别行政区基本法办事，全力支持香港、澳门两个特别行政区行政长官和政府依法施政，发展经济，改善民生，促进和谐。加强内地与港澳在经贸、科教、文化、卫生、体育等领域的交流与合作。

坚决反对任何谋求“台湾法理独立”的分裂活动

我们坚持“和平统一、一国两制”的基本方针，坚持新形势下发展两岸关系、推动祖国和平统一进程的各项政策。团结广大台湾同胞，坚决反对“台湾法理独立”等任何形式的分裂活动。

我们牢牢把握两岸关系和平发展主题，积极扩大两岸交流与合作，促进直接“三通”，以最大的诚意、尽最大的努力为两岸同胞谋和平、谋发展、谋福祉。我们将继续在一个中国原则基础上，加强同主张发展两岸关系的台湾各党派的对话和交流，争取早日恢复两岸对话与谈判，努力推动两岸关系朝着和平稳定方向发展。我们坚信，在包括台湾同胞在内的全体中华儿女的共同努力下，中国的完全统一一定能够实现！

为推动建设一个持久和平、共同繁荣的和谐世界而奋斗

我们愿与各国人民一道，为推动建设一个持久和平、共同繁荣的和谐世界而不懈努力！一年来，我们统筹国际、国内两个大局，积极倡导建设和谐世界，推动对外关系全面发展，为维护世界和平、促进共同发展做出了新贡献。

我们提出推动建设和谐世界，符合当今世界发展潮流和各国人民的共同利益与愿望，体现了中国政府和人民致力于世界和平与进步的坚定信念。建设和谐世界，就是要在政治上平等民主，经济上互利合作，文化上交流共进，通过国与国之间的友好合作，共同应对全球性的传统和非传统安全挑战，实现世界的持久和平与共同繁荣。面对复杂多变的国际形势，我们要高举和平、发展、合作的旗帜，坚定不移地走和平发展道路，坚定不移地奉行独立自主的和平外交政策，坚定不移地在和平共处五项原则基础上同世界各国和睦相处，捍卫国家的主权、安全和利益，维护我国发展的重要战略机遇期，为全面建设小康社会、加快社会主义现代化营造良好的外部环境。

我们要大力开展经贸、科技、文化、教育、体育等领域的对外交往与合作，增进同世界各国人民的了解和友谊，树立中国和平、民主、文明、进步的形象，维护我国公民和法人在海外的合法权益，尊重和依法保护其他国家公民在中国的合法权益。
